# Exploring health literacy and preferences for risk communication among medical oncology patients

**DOI:** 10.1371/journal.pone.0203988

**Published:** 2018-09-18

**Authors:** Mariko Carey, Anne Herrmann, Alix Hall, Elise Mansfield, Kristy Fakes

**Affiliations:** 1 Health Behaviour Research Collaborative, School of Medicine and Public Health, Faculty of Health and Medicine, University of Newcastle, Callaghan, NSW, Australia; 2 Priority Research Centre for Health Behaviour, University of Newcastle, Callaghan, NSW, Australia; 3 Hunter Medical Research Institute, New Lambton Heights, NSW, Australia; Boston University School of Medicine, UNITED STATES

## Abstract

**Objective:**

To explore adult medical oncology outpatients’ understanding of and preferences for the format of health risk information.

**Methods:**

Two surveys, one assessing sociodemographic characteristics and a second survey examining perceptions of risk information.

**Results:**

Of the 361 (74%) consenting patients, 210 completed at least one question on risk communication. 17% to 65% of patients understood numeric risk information, depending on the format of the information. More than 50% of people interpreted a “very good” chance of remission as greater than 80%, greater than 90% or 100%. The most preferred format of information was in both words and numbers (38% to 43%) followed by words alone (28% to 30%).

**Conclusion:**

Numeric risk information is understood by 17% to 65% of respondents, depending on the format. Interpretation of verbal risk information is highly variable, posing a risk of misunderstanding. Provision of information in both words and numbers may assist in aiding comprehension.

## Introduction

### Communication of risk is essential to assisting informed decision making for people with cancer

Optimal cancer care is patient-centred, placing great emphasis on involving patients in their healthcare decisions [1]. To achieve this, healthcare providers need to communicate accurate and unbiased health information to patients. However, this can be challenging as many cancer patients have a number of treatment options available to them, and the outcomes associated with each of these are probabilistic, leading to ambiguity and uncertainty [2]. In order to decide upon a treatment, patients often have to weigh-up uncertain risks against uncertain benefits of the treatment options available to them [3].

The way in which risks are presented can influence patient decision making [4]. For example, it has been suggested that patients often overestimate risk if it is presented as relative (e.g. your risk is two times higher than) rather than absolute risk (e.g. your risk is 5%) [5]. Therefore, risk and benefit information needs to be presented in a way that facilitates comparison across treatment options. This allows patients to integrate this information with their personal preferences and make informed decisions about their care [6].

### What format should risk be communicated in to optimise comprehension?

Patients vary in how they understand risks. For example, there is evidence to suggest that women with low literacy skills are more likely to overestimate their risk of developing breast cancer, compared to women with high literacy skills [7]. Numerous studies have looked at how to best present risks to patients [8, 9]. For example, it has been suggested that risk can be presented in form of graphs, verbal or numerical formats. Understanding of graphical risk presentation, such as icons or curves, may be influenced by the amount of instruction given and patients’ expertise [10]. There is considerable evidence to suggest that patients understand probabilistic information better if it is presented in numbers rather than words [11]. This may be because doctors and patients are likely to have different interpretations of what phrases like “low risk”, “unlikely”, or “high risk” mean [4]. Numbers are perceived to be precise, leading to more accurate perceptions of risk than the use of probability phrases and graphical displays [5]. Studies suggest that numeric probabilities associated with descriptors of risk such as “low” or “high” risk might increase comprehension of risk [12–14].

There are several ways of presenting numerical risk information, including as percentages, odds, or natural frequencies. It has been suggested that risks should be presented as natural frequencies with a small denominator (e.g. 1 out of 10) [5, 15]. Also, presenting numerical risks based on individual estimates, i.e. based on each individual patient’s characteristics, seems to be more effective in changing patient knowledge, attitudes, and behaviours than presenting risks based on general estimates [8, 16].

### What format do patients prefer risk to be communicated in?

Patients vary considerably in how they would like risk information to be presented to them [17]. While most patients prefer risks presented in numerical format rather than words [4, 5], this varies depending on sociodemographic characteristics, such as age, gender or educational level, as well as health status [17–19]. Findings from previous cross-sectional studies indicate that a range of complementary formats, including verbal and numerical description of risk, might be more appreciated by patients than the use of one format only [20–22].

Despite the increasing research effort in the area of risk communication, previous studies have to be considered in the light of several limitations. For example, many studies have been conducted with healthy people and findings might not be generalizable to people with serious medical conditions. There is little empirical data to guide our understanding of how adjectives should be used when communicating probabilities to people with cancer. Also, most studies in this area have involved recruitment of participants from just one clinic or hospital, and findings may not be applicable to all people with cancer. Further, most research has been conducted in the US [23, 24] and results may not be generalizable to other populations. Little is known about how Australian cancer patients understand different risk formats, and the way in which they want to be informed about the risks they face [19, 25].

## Aims

To explore, among medical oncology outpatients: 1) their understanding of numerical risk information and interpretation of adjectives used to describe risk; 2) their preferences for format of risk communication; and 3) characteristics associated with patients misunderstanding of risk-related information and preferences for receiving risk-related information in both written and number form.

## Methods

### Setting

The study was conducted as part of a larger study exploring psychological outcomes among medical oncology outpatients. Questions about risk communication were administered to participants recruited from two of the medical oncology clinics participating in the larger study. Both clinics were located in metropolitan public hospitals in Queensland and South Australia. The study was approved by the University of Newcastle Human Research Ethics Committee (H-2010-1324) as well as ethics committees associated with each participating institution.

### Participants

Medical oncology outpatients with a diagnosis of cancer, aged 18 or older and with sufficient English to complete the survey independently were eligible to participate.

### Procedure

Patients attending medical oncology outpatient clinics were invited to participate in the study. Informed written consent was obtained from all participants. Participants were asked to complete two pen-and-paper surveys. The initial survey was either completed in the clinic at the time of recruitment or taken home and mailed back to the researchers within one week. The second survey was mailed to the person’s home approximately one month later. For both surveys, reminder letters were sent to non-responders at two weeks. A second reminder letter was sent after four weeks of non-response.

### Measures

The first survey contained questions on sociodemographic, disease and treatment characteristics; while the second survey contained questions on understanding of and preferences for risk information.

#### Sociodemographic characteristics

Participants were asked to report age, gender, highest level of education, postcode, marital status, and whether or not they had a health care card or veterans' affairs card and / or private health insurance. A concession card is a government issued card that enables access to health services and medicines at a lower cost.

#### Disease and treatment characteristics

Type of cancer, time since diagnosis, stage of cancer, treatments undertaken for cancer and reason for outpatient consultation were assessed.

#### Understanding of numerical risk information

Respondents were given three questions about their understanding of numerical risk information: 1) “If a certain cancer drug is said to have a 30% chance of long-term side-effects, which statement is true?“; 2) “If you are told that a cancer treatment has a 5% risk of serious complications, which of the following are true?”; and 3) “If you are told that 1 in 5 people will experience a short-term side-effect from a cancer treatment, which of the following is correct?” Multiple response options were provided for each question and respondents were asked to select all that applied.

#### Interpretation of adjectives to describe risk

Respondents were asked, “If you were told that your chances of remission (i.e. being cancer free) were ‘very good’, what would you guess your chances of remission were?” Response options included: “more than 20%”; “more than 30%”; “more than 40%”; “more than 50%”: “more than 60%”: “more than 70%”; “more than 80%”; “more than 90%” and “100%”. Respondents were asked to select one response only.

#### Preferences for risk communication

Respondents were asked three questions about their preferences for information on likelihood of side-effects, remission and survival. For example: “Your doctor is telling you about your chances of long-term side-effects. How would you like your doctor to describe your chances of having long-term side-effects?” Response options included “in words (e.g. “poor”, “good”, “very good”)”; “in numbers (e.g. “three out of every ten people”)”; “in both words and numbers”; “I don’t care how my doctor gives me this information”; “I don’t want my doctor to give me this information”.

### Statistical analyses

All statistical analyses were conducted using SAS v9.4 (SAS Institute, Cary, North Carolina, USA). Descriptive statistics, including frequencies and percentages were calculated to answer each of the first two aims. Multivariable logistic regression analysis was conducted to identify patient sociodemographic characteristics associated with participants selecting only an incorrect response for at least one of the health literacy questions, as well as those who indicated a preference for receiving information in both words and numbers on topics covering: a) chances of long term side effects; b) chances of remission and c) chances of 5 year survival. Patients who indicated that they would prefer their doctor did not provide them with details concerning the risk-related topics were coded as missing for the multivariable analysis. The following characteristics were hypothesised as possibly being related to health literacy outcomes and were included in all logistic regression models: age, sex, education, concession card status and country of birth. For each characteristic assessed the unadjusted and adjusted odds ratios and the corresponding 95% confidence intervals (CIs) are presented, along with the Wald *p*-value for the final multivariable model.

## Results

608 people were screened for eligibility. Of these 117 were ineligible, and of the remaining 491, 361 (74%) agreed to take part in the study. Of those who consented, 217 returned a copy of the second survey, of which 210 completed at least one question on risk communication and were thus included in this analysis. There were no significant differences between non-consenters and study participants in terms of sex. However, there was a significant difference between non-consenters and study participants with respects to age (*p* = 0.02). Compared to non-consenters, there was a lower percentage of study participants aged less than 45 years (22% vs. 13%) and 65 years and over (36% vs. 30%); while there was a higher percentage of study participants aged between 45 and 64 years compared to non-consenters (57% vs. 42%).

Participant demographic and disease information are presented in Table 1. Almost half of participants were aged 60 years or over. Most were female, in a married or partnered relationship and were born in Australia. The most common cancer type was breast, with most cancers in the early stages. The main reason patients were visiting the treatment centre was to receive treatment.

**Table 1:** Sociodemographic characteristics of the sample (n = 210).

Characteristics	%	n
Age	3.3	7
Missing		
<45 years	12.9	27
> = 45 and <60 years	38.6	81
> = 60 years	45.2	95
Sex	2.9	6
Missing		
Male	20.5	43
Female	76.7	161
Marital status	3.8	8
Missing		
Married/partner	63.8	134
Single, divorced, separated or widowed	32.4	68
Education level	3.3	7
Missing		
High school or below	52.9	111
Trade, vocational training or University	39.5	83
Other	4.3	9
Country of birth	3.3	7
Missing		
Australia	67.6	142
Other	29.0	61
Health insurance	3.3	7
Missing		
Yes	19.5	41
No	77.1	162
Concession card	3.8	8
Missing		
Yes	54.3	114
No	41.9	88
Cancer type	4.3	9
Missing		
Breast	47.1	99
Colorectal	7.6	16
Lung	4.8	10
Other	36.2	76
Time since diagnosis	3.3	7
Missing		
0–6 months	25.7	54
7–12 months	17.6	37
13–24 months	15.7	33
24+ months	37.6	79
Cancer stage	6.2	13
Missing		
Early	56.2	118
Progressed/advanced	30.0	63
NA or don't know	7.6	16
Reason for visit	5.2	11
Missing		
To discuss treatment options	6.7	14
To receive treatment	43.8	92
To have a check-up during treatment	15.7	33
To have a check-up after treatment	24.3	51
Other	4.3	9

### Understanding of risk information

204 participants provided a response to the question: “If a certain cancer drug is said to have a 30% chance of long term side-effects, which statement is true?” Of these, most participants (n = 125; 61%) endorsed only the correct response: “3 out of every 10 people who take this drug will have long term side-effects.” A smaller number of people endorsed the correct response and one or more incorrect responses (n = 18; 8.8%), with the remaining respondents endorsing incorrect responses only (n = 61; 30%).

203 participants answered the question: “If you are told that a cancer treatment has a 5% risk of serious complications, which of the following are true?” For this question, two responses were considered correct: “The risk of complications is low but I am still at risk” was the most frequently endorsed correct response (endorsed by n = 154; 76%) and “50 out of 1000 people will experience this complication” (endorsed by n = 74; 36%). Thirty-five (17%) endorsed both correct answers, while 137 (67%) endorsed one of the correct responses only. A further 15 (7.4%) respondents selected at least one of the correct responses together with an incorrect option, and sixteen participants (7.9%) selected only incorrect responses.

A total of 199 participants answered the question: “If you are told that 1 in 5 people will experience a short-term side-effect from a cancer treatment, which of the following is correct?” Of these respondents, 130 (65%) endorsed only the correct response, which was “The risk of experiencing short-term side-effects from this treatment is 20%”. A small number endorsed both the correct response and at least one incorrect response (n = 6; 3.0%), while almost a third selected only incorrect responses (n = 63; 32%). Of the incorrect responses the most frequently endorsed by participants was “The risk of experiencing short-term side-effects from this treatment is 5%” (n = 43; 22%).

### Characteristics associated with participants selecting an incorrect response

A total of 193 participants had complete data on all variables included in the logistic regression model for this outcome and were thus included in this analysis. 87 patients reported only an incorrect response on at least one of the three health literacy items. As shown in Table 2, after adjusting for all other characteristics education level was the only characteristic found to be significant. Compared to participants who had a trade, vocational training, university or other level of education, those with an education level of high school or below had more than twice the odds of reporting only an incorrect response on at least one of the health literacy items.

**Table 2:** Characteristics associated with patients selecting an incorrect response only, on at least one of the health literacy items.

Characteristic	Category	Unadjusted OR (95% CI)	Adjusted OR (95% CI)	Adjusted p-value
Age	< 45 years vs. 60+ years	0.45 (0.18, 1.15)	0.73 (0.26, 2.07)	0.6878
	45 to 59 years vs. 60+ years	0.71 (0.39, 1.30)	1.13 (0.53, 2.39)	
Concession card	Yes concession card vs. No concession card	2.09 (1.17, 3.74)	1.96 (0.99, 3.87)	0.0525
Country of birth	Country other than Australia vs. Australia	1.09 (0.59, 2.01)	1.18 (0.61, 2.29)	0.6144
Education level	High school or below vs. Trade, vocational training, University or Other	2.70 (1.50, 4.85)	2.58 (1.36, 4.88)	0.0037
Sex	Female vs. Male	1.39 (0.69, 2.81)	1.75 (0.82, 3.76)	0.1500

### Interpretation of adjectives to describe risk

**Fig 1:**
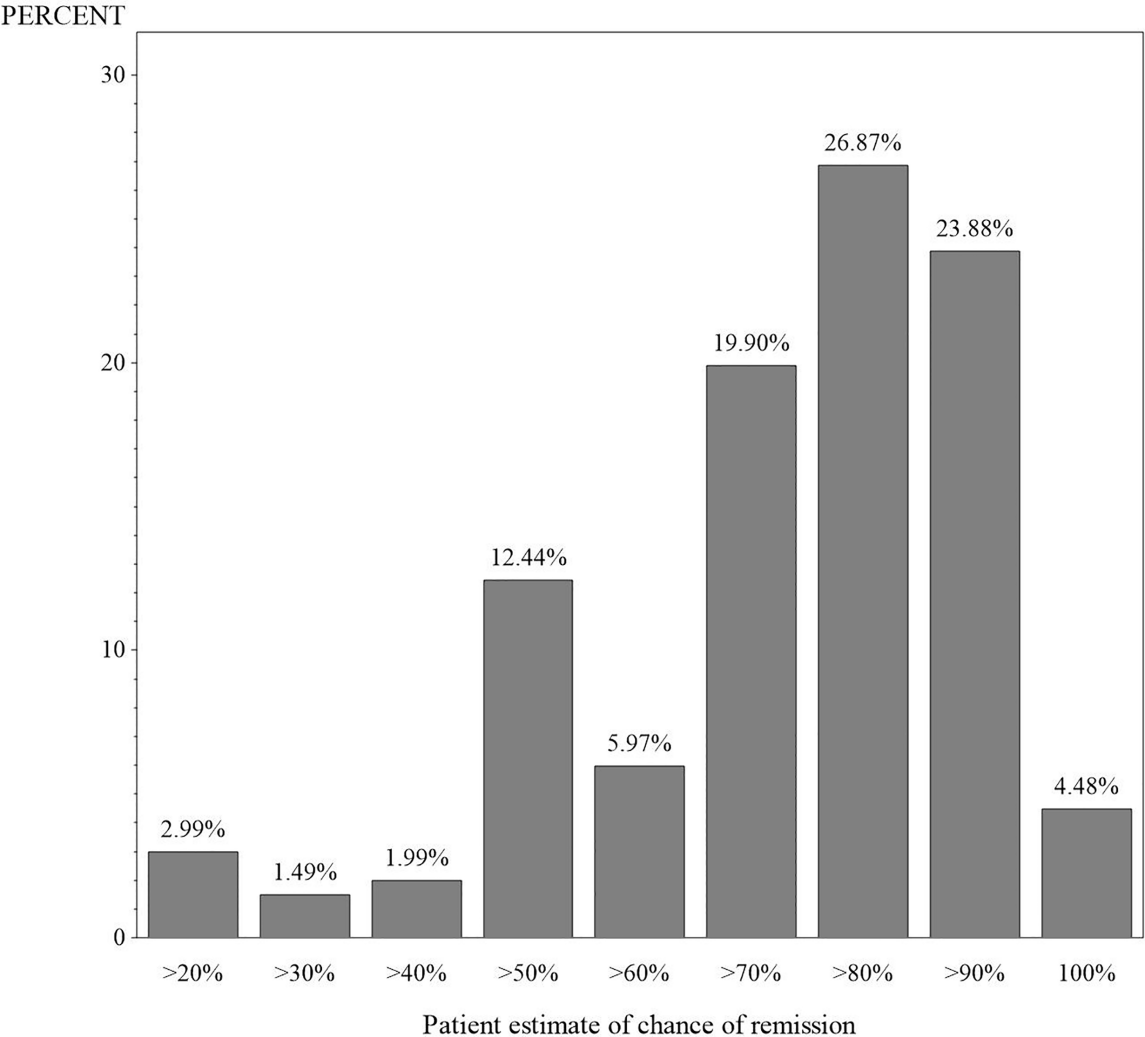
Participants’ interpretation of a “very good” chance of remission as a percentage.

Two hundred and one participants provided an answer to the question: “If you were told that your chances of remission (i.e. being cancer free) were ‘very good’, what would you guess your chances of remission were?” The results of participant responses are presented in Fig 1. The most common estimate was “more than 80%” with 54 (27%) of respondents selecting this option. This was closely followed by “more than 90%”, which was endorsed by 48 (24%) of respondents. The least frequent response option selected by respondents was “more than 30%” (n = 3; 1.5%).

### Preferences for format of health risk communication

When participants were asked how they would like to have their chances for long-term side-effects, remission and five-year survival communicated to them, the most frequently reported preference for all three topics was for both words and numbers to be used (Table 3). Words alone was the second most frequently endorsed option across all three topics, while only a small minority of patients indicated a preference for not being told about their chances at all.

**Table 3:** Preferences for health risk communication.

Preferred format of communication	Chances of long-term side-effects	Chances of remission	Chances of five year survival
Missing	5 (2.4%)	5 (2.4%)	8 (3.8%)
Words	59 (28%)	62 (30%)	58 (28%)
Numbers	33 (16%)	21 (10%)	17 (8.1%)
Both words or numbers	79 (38%)	88 (42%)	90 (43%)
Don't care	33 (16%)	33 (16%)	31 (15%)
Prefer not to be told	1 (0.5%)	1 (0.5%)	6 (2.9%)

### Characteristics associated with patients preferring information in both words and numbers

Table 4 describes the crude and adjusted logistic regression results assessing characteristics associated with patient preference for information to be presented in both numbers and words for the following topics: a) chances of long-term side effects; b) chances of remission and c) chances of 5-year survival. For each of the three models a total of 195, 195 and 187 participants had complete data on all variables included in the logistic regression model, respectively, and were thus included in the analysis. As shown in Table 4, after adjusting for all other characteristics, age was the only characteristic found to be significantly associated with the three outcomes assessed. For all three outcomes, compared to participants who were aged 60 years or over, participants who were aged between 45 and 59 years had significantly higher odds of preferring cancer-related risk information to be presented to them in both numbers and words.

**Table 4:** Characteristics associated with patients preferring information to be presented in both words and numbers.

Risk topic	Characteristic	Category	Unadjusted OR (95% CI)	Adjusted OR (95% CI)	Adjusted p-value
Chances of long-term side-effects	Age	<45 years vs. 60+ years	2.75 (1.13, 6.69)	2.34 (0.87, 6.32)	0.033
		45 to 59 years vs. 60+ years	3.36 (1.76, 6.41)	2.73 (1.27, 5.87)	
	Concession card	Yes concession card vs. No concession card	0.58 (0.32, 1.03)	1.03 (0.52, 2.06)	0.929
	Country of birth	Country other than Australia vs. Australia	1.18 (0.63, 2.19)	1.38 (0.70, 2.73)	0.350
	Education level	High school or below vs. Trade, vocational training, University or Other	0.49 (0.27, 0.87)	0.67 (0.35, 1.26)	0.213
	Sex	Female vs. Male	2.91 (1.30, 6.48)	2.23 (0.96, 5.22)	0.063

Chances of remission	Age	<45 years vs. 60+ years	2.75 (1.13, 6.69)	1.97 (0.72, 5.36)	0.007
		45 to 59 years vs. 60+ years	4.83 (2.51, 9.30)	3.44 (1.60, 7.41)	
	Concession card	Yes concession card vs. No concession card	0.38 (0.21, 0.68)	0.73 (0.36, 1.47)	0.380
	Country of birth	Country other than Australia vs. Australia	1.16 (0.63, 2.15)	1.25 (0.62, 2.51)	0.539
	Education level	High school or below vs. Trade, vocational training, University or Other	0.39 (0.22, 0.69)	0.53 (0.27, 1.01)	0.054
	Sex	Female vs. Male	3.09 (1.43, 6.71)	2.17 (0.94, 5.01)	0.069

Chances of five year survival	Age	<45 years vs. 60+ years	2.56 (1.04, 6.28)	2.19 (0.80, 6.01)	0.002
		45 to 59 years vs. 60+ years	4.73 (2.43, 9.18)	4.15 (1.88, 9.15)	
	Concession card	Yes concession card vs. No concession card	0.52 (0.29, 0.93)	1.10 (0.53, 2.26)	0.804
	Country of birth	Country other than Australia vs. Australia	1.06 (0.57, 1.98)	1.16 (0.57, 2.32)	0.686
	Education level	High school or below vs. Trade, vocational training, University or Other	0.48 (0.27, 0.86)	0.64 (0.33, 1.24)	0.187
	Sex	Female vs. Male	3.23 (1.48, 7.05)	2.25 (0.97, 5.23)	0.059

## Discussion

This study is one of few to examine the understanding and interpretation of health risk information and format preferences in Australian medical oncology outpatients. Overall, our findings revealed that in relation to numerical risk information, natural frequencies with a small denominator were the most understood format. However, in relation to risk wording, a wide variation in patient interpretations was found. The use of both words and numbers was the most frequently patient-preferred format for risk communication.

When numerical risk information was presented simply using natural frequencies and a small denominator (e.g. “3 out of every 10 people”), approximately 61% of people understood the information. Of the 32% who selected an incorrect response regarding their understanding of the term “1 in 5 people…”, almost a quarter of respondents (22%) perceived that the risk meant a 5% rather than 20% risk, indicating that misunderstandings may be significant in some cases. When presented with a larger denominator (e.g. “50 out of 1000 people”), the number of people who understood the information reduced to about one third (36%). This supports previous findings that information presented with a small denominator are more likely to be understood and should be used to present risk information to patients [9]. Similar to other studies [26–28], our results also suggests that those with lower levels of education have higher odds of misinterpreting risk information.

Our findings revealed a wide variation in the way that patients interpreted risk adjectives. Participant interpretations of what a “very good” chance of remission ranged from “more than 20%” to “100%”. It is notable that 28% of participants perceived that “very good” meant a 90% or greater chance; while over half the sample (55%) perceived that this meant 80% or greater. This suggests that there is great potential for misunderstanding where only verbal risk descriptors are used. The wide variation in responses is consistent with previous research which has found that patients more accurately understand risk information if it is presented in numbers rather than words [9].

The most frequently reported preference for the format of information regarding long-term side-effects, remission, and five-year survival was for both words and numbers to be used, at 38%, 42% and 43%, for each of the three risk topics, respectively. The second most frequently endorsed option was for words alone to be used, which was endorsed by approximately 30% of respondents across all three risk topics. While patients vary in how they would like risks to be presented to them [17], our results differ from other research which has suggested that most patients prefer risks to be presented in a numerical format [4, 5, 17–19]. However, most prior research has not been conducted in an oncology setting, and patients’ preferences may be affected by illness severity, health status and other sociodemographic factors [4]. Our results, for example, suggested that younger patients had higher odds of preferring information in both words and numbers than older patients.

Our findings must be considered in view of several limitations. Firstly, participants were recruited from three medical oncology clinics, and so are unlikely to be representative of Australian medical oncology patients. While a consent rate of 74% was achieved, there were a number of participants who did not return the second survey that contained the health literacy questions. This may have further impacted on generalisability of results. When asking participants about their understanding of a 5% risk of serious complications, we counted the following response as one of two possible correct answers: “The risk of complications is low, but I am still at risk”. We acknowledge that interpretation of 5% risk as “low” is subjective and could be debated.

### Conclusion

Our results suggest that risk information presented in natural frequencies with small denominators (e.g. 1/5) is understood by 61%-65%, depending on the scenario presented. When risk information is presented with large denominators, a lower proportion indicate that they understand (36%). We found substantial variation in patients’ interpretation of risk descriptors (e.g. “good”, “very good”), highlighting the dangers of providing patients with risk descriptors without accompanying numeric information. Patients were most likely to have a preference for receiving risk information as both words and numbers.

### Practice implications

The findings provide guidance as to how physicians should communicate risk information about outcomes of treatment such as possible side-effects, and likelihood of remission and survival. The variation in patents’ interpretation of risk information when this is presented in words only may result in unrealistic expectations regarding outcomes. To overcome this variation, risk information in words should be combined with risk information in numbers. Together with other studies [9, 29], our findings suggest that numeric information should be presented as natural frequencies with small rather than large denominators to aid patient understanding.

However, it is important to note that a large proportion of patients (just under one third) did not understand the risk information when it was presented in this ‘optimal’ format. This suggests that physicians should probe patient understanding of risk, and utilise other formats to supplement this information if necessary. This could include, for example, diagrams showing the number of people out of 10 who are likely to experience a certain outcome, or bar charts showing the proportion of patients likely to experience each outcome. Graphical formats have been shown to improve patients’ understanding of risk information [9], and may also reduce participants’ reliance on anecdotal evidence when making decisions [30]. The use of additional strategies to aid patient comprehension of risk information may be particularly important when presenting information to patients who have a lower level of education.

## Supporting information

S1 Survey (DOCX)
