# Supplementary material for: Exploring health literacy and preferences for risk communication among medical oncology patients
Source: PLoS One. 2018 Sep 18;13(9):e0203988. doi: 10.1371/journal.pone.0203988 (PMC6143261; doi:10.1371/journal.pone.0203988)
Supplement: S1 Survey — (DOCX) [file pone.0203988.s001.docx]

**Communication of medical information**

Your doctor may give you information about your cancer treatment. For example, your doctor may tell you about your chances of experiencing different side effects of treatment. This type of information can be presented in different ways. We are interested in your understanding of this type of information. We are also interested in how you would like this information to be given.

**Please answer all the items in this module. For each of the following questions please select the option which you believe is correct.**

| **1.** | **If a certain cancer drug is said to have a 30% chance of long-term side-effects, which statement is true?**  *Please choose* ***all*** *answers you believe to be correct* | 1. 3 out of every 10 people who take this cancer drug will have long-term side-effects 2. 30 out of every 1000 people who take this cancer drug will have long-term side-effects 3. 70 out of 1000 people who take this cancer drug will not have long-term side-effects 4. 1 out of 3 people who take this cancer drug will not have long-term side-effects 5. None of the above |
| --- | --- | --- |
| **2.** | **If you are told that a cancer treatment has a 5% risk of serious complications, which of the following are true?**  *Please choose* ***all*** *answers you believe to be correct* | - 1. The risk of complications is low, so I am not at risk   2. Only 1 out of every 5 people will experience a complication   3. The risk of complications is low, but there is still a chance that I am at risk   4. 50 out of every 1000 people will experience this complication   5. None of the above |
| **3.** | **If you are told that 1 in 5 people will experience a short-term side-effect from a cancer treatment, which of the following is correct?**  *Please choose* ***all*** *answers you believe to be correct* | 1. The risk of experiencing short-term side-effects from this treatment is 5% 2. The risk of experiencing short-term side-effects from this treatment is 50% 3. The risk of experiencing short-term side-effects from this treatment is 20% 4. The risk of experiencing short-term side-effects from this treatment is 15% 5. None of the above |
| **4.** | **If you were told that your chances of remission (i.e. being cancer free) were ‘very good’, what would you guess your chances of remission were?**  *Please choose one answer only* | 1. More than 20% 2. More than 30% 3. More than 40% 4. More than 50% 5. More than 60% 6. More than 70% 7. More than 80% 8. More than 90% 9. 100% |
| **5.** | **Your doctor is telling you about your chances of long-term side-effects. How would you like your doctor to describe your chances of having long-term side-effects?**  *Please choose one answer only* | 1. In words, such as “unlikely/possible/highly likely” 2. In numbers, such as “3 out of every 10 people will experience long-term side-effects from using this cancer treatment” 3. Both words or numbers 4. I do not care how my doctor explains this information to me 5. I would prefer my doctor did not tell me this information |
| **6.** | **Your doctor is explaining to you your chances of your cancer going into remission (i.e. being free from cancer). How would you like your doctor to describe your chances of your cancer going into remission?**  *Please choose one answer only* | 1. In words, such as “poor/good/very good” 2. In numbers, such as “3 out of every 10 people diagnosed with your cancer will experience remission” 3. Both words or numbers 4. I do not care how my doctor explains this information to me 5. I would prefer my doctor did not tell me this information |
| **7.** | **Your doctor is explaining to you your chances of surviving from your cancer diagnosis in the next five years. How would you like your doctor to explain to you this information?**  *Please choose one answer only* | - 1. In words, such as “poor/good/very good.”   2. In numbers, such as “3 out of every 10 people diagnosed with your cancer will survive the first five years after being diagnosed”   3. Both words or numbers   4. I do not care how my doctor explains this information to me.   5. I would prefer my doctor did not tell me this information. |
